# Spontaneously Diabetic Torii Fatty Rat Shows Early Stage of Diabetic Retinopathy Characterized by Capillary Changes and Inflammation

**DOI:** 10.1155/jdr/3800292

**Published:** 2025-04-21

**Authors:** Kasumi Kikuchi, Miyuki Murata, Yasushi Kageyama, Masami Shinohara, Tomohiko Sasase, Kousuke Noda, Susumu Ishida

**Affiliations:** ^1^Laboratory of Ocular Cell Biology & Visual Science, Department of Ophthalmology, Faculty of Medicine and Graduate School of Medicine, Hokkaido University, Sapporo, Hokkaido, Japan; ^2^Tokyo Animal & Diet Department, CLEA Japan, Inc., Tokyo, Japan; ^3^Biological/Pharmacological Research Laboratories, Central Pharmaceutical Research Institute, Japan Tobacco Inc., Takatsuki, Osaka, Japan; ^4^Sapporo Sousei East Clinic, Sapporo, Hokkaido, Japan

**Keywords:** animal model, diabetic retinopathy, SDT fatty rat

## Abstract

**Purpose:** The Spontaneously Diabetic Torii (SDT) fatty rat is an animal model of obese Type 2 diabetes. We previously reported that the SDT fatty rats develop diabetic cataracts. This study aimed to elucidate early diabetic changes in the retina of the SDT fatty rats.

**Materials and Methods:** The retinal thickness, capillary diameter, and pericyte/endothelial cell (P/E) ratio were assessed in the male SDT fatty rats and Sprague–Dawley (SD) rats at 24 weeks of age. Immunostaining was performed to assess the intercellular adhesion molecule-1 (ICAM-1) and vascular cell adhesion molecule-1 (VCAM-1) levels in the retinal capillaries. DNA microarray analysis was performed to detect inflammation-associated molecules in the retina of the SDT fatty rats. Real-time PCR and Magnetic Luminex Assay were performed to validate the results.

**Results:** The retinal thickness in the SDT fatty rats was significantly greater than that in SD rats. The capillary diameter in the retina of the SDT fatty rats was significantly higher than that of SD rats. The P/E ratio in the SDT fatty rats was significantly lower than that in SD rats. ICAM-1 and VCAM-1 were observed in the retinal vessels of the SDT fatty rats. The levels of mRNA and protein of *Mcp1*, *Il1b*, *Icam1*, and *Tnf* were upregulated in the retinal tissues of the 24-week-old SDT fatty rats.

**Conclusions:** Our study demonstrated that the SDT fatty rats exhibited early diabetic retinal changes, suggesting that the SDT fatty rats may be useful in research on the pathogenesis of early human diabetic retinopathy.

## 1. Introduction

The prevalence of diabetes is on the rise globally. In 2021, approximately 536.6 million individuals were affected by diabetes, and this number is projected to increase to 783.2 million by 2045 [1]. One-third of adults with diabetes have diabetic retinopathy (DR), which is one of the primary causes of visual loss among those of working age [2]. Diabetic macular edema (DME) is a pathological thickening and swelling of the macula that results from microvascular complications [3]. It can develop at any stage of DR and can lead to visual impairment. Intravitreal injections of anti-vascular endothelial growth factor (VEGF) drugs, laser photocoagulation, and intravitreal or subtenon administration of steroids have been used in the treatment of DME [4]. However, these interventions are performed in cases presenting with advanced stage of DR. To maintain good visual function in the increasing number of patients with DR, it is imperative to implement early treatment strategies before the onset of retinal structural changes. This necessitates a comprehensive and nuanced understanding of the underlying mechanisms and pathogenesis of early DR.

The Spontaneously Diabetic Torii (SDT) fatty rat is an animal model of obese Type 2 diabetes. The SDT fatty rats exhibit hyperglycemia within 5 weeks. [5, 6] We previously reported that the SDT fatty rats exhibit cortical and posterior subcapsular cataracts that mimic human diabetic cataracts [6].

In this study, we investigated retinopathy in the SDT fatty rats and compared their characteristics with those of human DR to clarify the similarities.

## 2. Materials and Methods

### 2.1. Animals

Male SDT fatty rats and age-matched male Sprague–Dawley (SD) rats (CLEA Japan Inc., Tokyo, Japan) were housed in the animal facility at Hokkaido University, with free access to standard food and water. The experimental protocols were approved by the Animal Experimentation Committee of Hokkaido University (approval number: 18-0115).

### 2.2. Retinal Thickness Measurement

All rats were euthanized by an overdose of anesthetic at the age of 24 weeks. The enucleated eyes were stored in a fixative solution (Super Fix, Kurabo Industries Ltd., Osaka, Japan), embedded in paraffin, and sectioned using standard techniques. Hematoxylin and eosin (H&E) staining was used to observe retinal changes. We defined the total thickness in this study as the distance between the internal limiting membrane (ILM) and photoreceptor layer. The total retinal and inner nuclear layer (INL) thicknesses were measured at a distance of 1500 *μ*m from the optic nerve head.

### 2.3. Trypsin Digestion and H&E Staining

Trypsin digestion was used to evaluate the retinal capillaries. The retinal tissues were dissected from the eyeballs at 24 weeks of age and divided into four sections after fixation in 10% formalin neutral buffer solution (Fujifilm Wako, Osaka, Japan) for 24 h. The retinal sections were washed overnight with distilled water and incubated with 1% Triton X-100 (Merck, Darmstadt, Germany) for 15 min. The tissues were digested with 3% Trypsin (Thermo Fisher Scientific, Waltham, MA, United States) at 37°C for 45 min. The retinal vasculature, mounted and dried on slide glass, was stained using H&E. Photographs of the site at a distance of 1500 *μ*m from the optic nerve head were acquired, and the diameter of the capillary vessel was measured. The vessels within the two branches of the main vessels were excluded from the measurement.

### 2.4. Immunofluorescence Microscopy

Following trypsin digestion, immunofluorescence staining was conducted to detect the presence of leukocyte adhesion molecules. The trypsin-digested specimens were fixed in a precooled solution containing acetic acid and ethanol at a 1:2 ratio for 5 min at −20°C. The specimens were rinsed in phosphate-buffered saline and incubated with 10% goat serum (Thermo Fisher Scientific) for 30 min. The specimens were subsequently incubated with the following primary antibodies: rabbit polyclonal anti-NG2 (1:100, AB5320, Merck), mouse monoclonal anti-intercellular adhesion molecule-1 (ICAM-1; 1:100, 1A29, Novus biological, Centennial, CO, United States), and rabbit monoclonal anti-vascular cell adhesion molecule-1 (VCAM-1; 1:50, EPR5047, Abcam, Cambridge, United Kingdom). Goat anti-mouse immunogloblin G (IgG, H + L) Alexa Fluor plus 488 and goat anti-rabbit IgG (H + L) Alexa Fluor 546 (1:500; Thermo Fisher Scientific) were used as secondary antibodies for fluorescence detection. The nuclei were stained using 4⁣′,6-diamidino-2-phenylindole (DAPI) and viewed with a fluorescence microscope (BZ-9000, Keyence, Osaka, Japan).

All retinal layers were immunostained for glial fibrillary acidic protein (GFAP). The deparaffinization and hydration of paraffin sections were conducted by immersing them in xylene, alcohol, and water. Antigen retrieval was performed using microwave in 10 mM citrate buffer (pH 6.0), and the sections were incubated in 10% goat serum (Thermo Fisher Scientific) for 30 min. The sections were subsequently incubated overnight at 4°C in a solution containing a primary mouse monoclonal antibody against GFAP (1:50, GA5; Thermo Fisher Scientific). The sections were exposed to goat anti-mouse IgG (H + L) Alexa Fluor plus 488 (1:500; Thermo Fisher Scientific) for 1 h at room temperature.

### 2.5. Pericyte/Endothelial Ratio (P/E Ratio)

The retinal capillary network isolated via trypsin digestion was immunostained using an anti-NG2 antibody. The total number of cells in the blood vessels was enumerated and categorized into two groups: NG2-positive cells (pericytes) and NG2-negative cells (endothelial cells). The P/E ratio was subsequently calculated.

### 2.6. DNA Microarray

NucleoSpin RNA Plus (MACHEREY-NARGEL, Düren, Germany) was used to extract the total RNA from the retina of the SDT fatty and SD rats. Microarray analysis (SurePrint G3 Rat Gene Expression 8 × 60 K v2 Microarray; Agilent, Santa Clara, CA, United States) was performed by Takara Bio Inc. (Shiga, Japan).

### 2.7. Quantitative Real-Time Polymerase Chain Reaction (PCR)

Quantitative real-time PCR was conducted to ascertain the expression levels of *Mcp1*, *Il1b, Icam1, Tnf*, *Vegfa*, and *Il6* mRNA. NucleoSpin RNA Plus was employed for the extraction of total RNA from the retina of 5-, 8-, 12-, 16- or 24-week-old rats, which were reverse transcribed into cDNA, and mRNA levels were analyzed using GoTaq qPCR and RT-qPCR systems (Promega Fitchburg, WI, United States) on a StepOnePlus Real-Time PCR System (Thermo Fisher Scientific). The primer sequences utilized for real-time PCR and PCR product sizes were as follows: 5⁣′-CTGGGCCTGTTGTTCACAGTT-3⁣′ (forward) and 5⁣′-GTGAATGAGTAGCAGCAGGTGA-3⁣′ (reverse) for rat *Mcp1* (NM_031530), 95 bp; 5⁣′-GTCTGAAGCAGCTATGGCAAC-3⁣′ (forward) and 5⁣′-CATCTGGACAGCCCAAGTCAA-3⁣′ (reverse) for rat *Il1b* (NM_031512), 146 bp; 5⁣′-TGGTCCTCCAATGGCTTCAAC-3⁣′ (forward) and 5⁣′-GATGGATACCTGAGCACCGA -3⁣′ (reverse) for rat *Icam1* (NM_012967), 103 bp; 5⁣′-GGGTGATCGGTCCCAACAAG-3⁣′ (forward) and 5⁣′-CGCTTGGTGGTTTGCTACGA-3⁣′ (reverse) for rat *Tnf* (NM_012675), 143 bp; 5⁣′-AGCAGATGTGAATGCAGACCAAAGA-3⁣′ (forward) and 5⁣′-TGGCTCACCGCCTTGGCTT-3⁣′ (reverse) for rat *Vegfa* (NM_001287107), 205 bp; 5⁣′-AGCGATGATGCACTGTCAGA-3⁣′ (forward) and 5⁣′-GGAACTCCAGAAGACCAGAGC-3⁣′ (reverse) for rat *Il6* (NM_012589), 127 bp; and 5⁣′-GGGAAATCGTGCGTGACATT-3⁣′ (forward) and 5⁣′-GCGGCAGTGGCCATCTC-3⁣′ (reverse) for rat *Actb* (NM_031144), 76 bp. The PCR conditions used were as follows: 95°C for 2 min, followed by 40 cycles of 95°C for 15 s, and 60°C for 1 min. The *ΔΔ*Ct method was employed for calculations.

### 2.8. Magnetic Luminex Assay

Magnetic Luminex Assay (R&D systems, Minneapolis, MN, United States) was performed to assess the monocyte chemoattractant protein-1 (MCP-1), interleukin-1*β* (IL-1*β*), ICAM-1, tumor necrosis factor-*α* (TNF-*α*), VEGF-A, and interleukin-6 (IL-6) levels in the retina. Microbeads coated with the respective rat antibodies, retinal tissue lysate, and standards were placed in a 96-well plate and incubated. A biotin antibody cocktail was subsequently added. The plates were washed after 1 h of incubation, and streptavidin–phycoerythrin was subsequently added. The microbeads were incubated for 30 min. The plates were subjected to a final wash and resuspended in buffer solution. The plates were analyzed using the MAGPIX (Merck) and xPONENT software (Merck). All results were standardized to the protein concentrations according to the BCA protein assay kit (Thermo Fisher Scientific).

### 2.9. Statistical Analysis

The results are expressed as mean ± standard error of the mean, with sample sizes (*n*) specified. Differences in mean values between the two groups were analyzed using Student's *t*-test. A *p* value of < 0.05 was considered statistically significant.

## 3. Results

### 3.1. Morphological Changes in the Retina of SDT Fatty Rats

**Figure 1:**
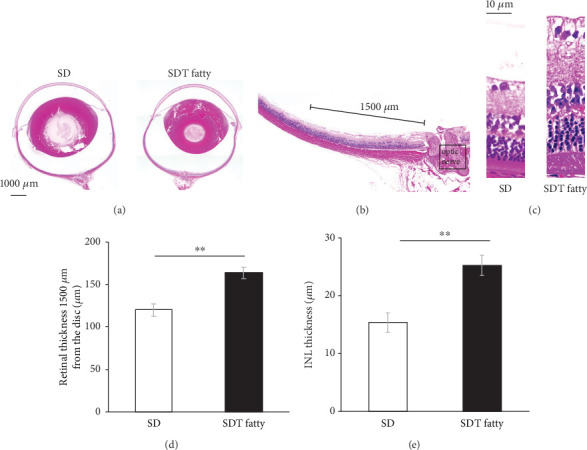
Retinal thickness in SDT fatty rats and SD rats. Histopathological findings in the retina of SDT fatty rats and SD rats. (a) Representative microscopic image of hematoxylin and eosin (H&E)-stained eyeballs from 24-week-old male SD rats and SDT fatty rats. Index bar = 1000*  μ*m. (b, c) The retinal thickness was measured at a distance of 1500 *μ*m from the optic nerve head. (d) In SDT fatty rats, the total retinal thickness was found to be significantly greater than that in SD rats. *n* = 8, each. ⁣^∗∗^*p* < 0.01. (e) The INL thickness in SDT fatty rats was significantly higher than that in SD rats. *n* = 8, each. ⁣^∗∗^*p* < 0.01. SDT, Spontaneously Diabetic Torii; SD, Sprague–Dawley; INL, inner nuclear layer.

**Figure 2:**
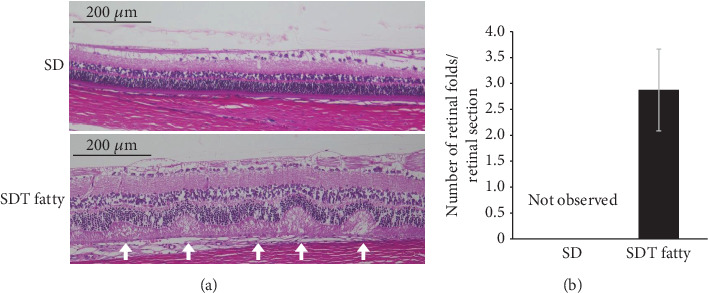
Retinal folds in SDT fatty rats and SD rats. (a) Retinal folds were observed in retinal specimens from 24-week-old SDT fatty rats, while no retinal folds were detected in SD rats. (b) The mean number of retinal folds in SDT fatty rats was 2.88 ± 0.79/retinal section. SDT fatty rats, *n* = 8; SD rats, *n* = 7. SDT, Spontaneously Diabetic Torii; SD, Sprague–Dawley.

The histological findings in the H&E-stained tissue sections were assessed to determine whether diabetic changes were observed in the retina of the SDT fatty rats. The retina of the SDT fatty rats were observed to have thickened compared with that of the SD rats (Figure 1a). High-magnification images obtained at a distance of 1500 *μ*m from the optic nerve head revealed thickening of all layers, particularly the INL, in the retinal tissue of the SDT fatty rats (Figure 1b,c). Quantitative analysis of the area 1500 *μ*m from the optic nerve head revealed that the total retinal thickness in the SDT fatty rats (163.13 ± 6.79*  μ*m, *n* = 8) was significantly higher than that in SD rats (119.75 ± 7.35*  μ*m, *n* = 8, *p* < 0.01; Figure 1d). The thickness of INL was also significantly higher in the SDT fatty rats (25.16 ± 1.74*  μ*m, *n* = 8) than in SD rats (15.44 ± 1.64*  μ*m, *n* = 8 each, *p* < 0.01; Figure 1e). In addition, retinal folds were observed in seven out of eight retinas of the 24-week-old SDT fatty rats, whereas no retinal folds were observed in SD rats. The mean number of retinal folds in the 24-week-old SDT fatty rats was 2.88 ± 0.79/retinal section (Figure 2a,b).

### 3.2. Microvascular Alterations in SDT Fatty Rats

**Figure 3:**
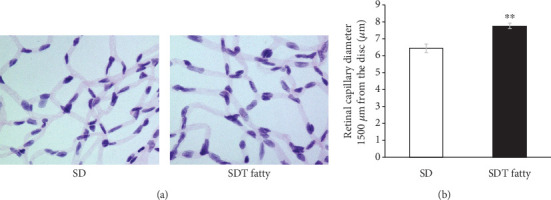
Analysis of the retinal blood vessels. (a) Representative of trypsin-digested blood vessels (H&E stain). (b) The capillary diameter of SDT fatty rats was significantly higher than that of SD rats. *n* = 5, each. ⁣^∗∗^*p* < 0.01. SDT, Spontaneously Diabetic Torii; SD, Sprague–Dawley.

**Figure 4:**
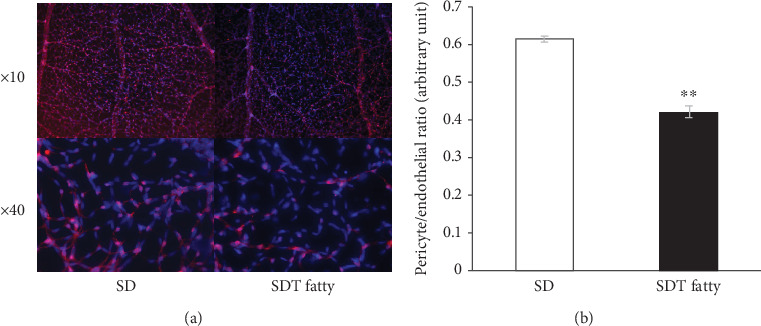
Pericyte/endothelial ratio in SDT fatty rats. (a) Representative photo of the immunostained retinal vessels. NG-2 (red) and DAPI (blue). (b) The P/E ratio in SDT fatty rat retina showed a significant decrease. *n* = 5, each. ⁣^∗∗^*p* < 0.01. SDT, Spontaneously Diabetic Torii; DAPI, 4⁣′,6-diamidino-2-phenylindole; P/E ratio, pericyte/endothelial ratio.

**Figure 5:**
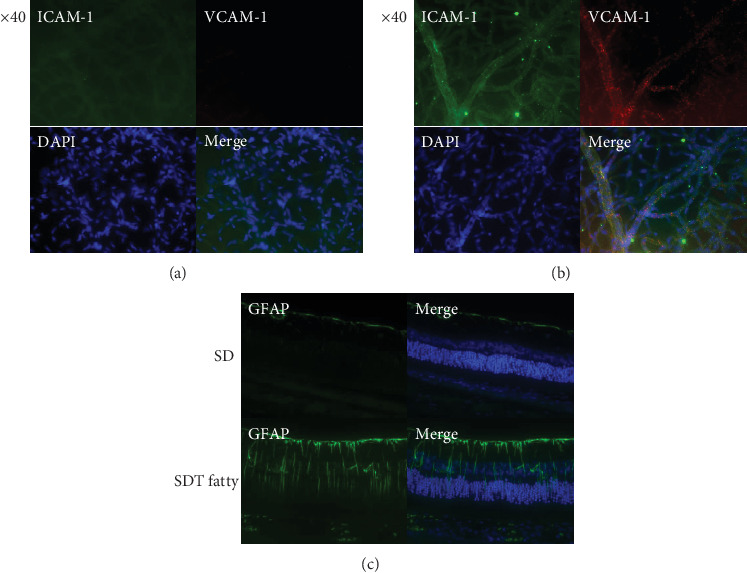
ICAM-1, VCAM-1, and GFAP expressions in the retina of the rats. (a) Representative photo of immunostained retinal vessels of SD rats. The retinal vessels of SD rats did not express ICAM-1 (green) or VCAM-1 (red). (b) The retinal vessels of SDT fatty rats express ICAM-1 (green) and VCAM-1 (red). (c) GFAP (green) immunofluorescence in the retinal sections. SDT, Spontaneously Diabetic Torii; SD, Sprague–Dawley; ICAM-1, intercellular adhesion molecule-1; VCAM-1, vascular cell adhesion molecule-1; GFAP, glial fibrillary acidic protein.

Microvascular alterations, such as capillary dilation, microaneurysm formation, and pericyte loss, are hallmark features of DR. Therefore, structural changes in the retinal capillaries of the SDT fatty rats were first evaluated. The capillary diameter in the SDT fatty rats (7.74 ± 0.16*  μ*m, *n* = 5) was significantly larger than that in the SD rats (6.42 ± 0.23*  μ*m, *n* = 5, *p* < 0.01; Figure 3a,b). Microaneurysm formation was not observed in the retina of the 24-week-old SDT fatty rats. The distribution of NG2-positive cells in the retinal vessels of the SDT fatty and SD rats was examined to determine whether pericytes were damaged in the retinal vessels of the SDT fatty rats (Figure 4a). Fewer NG2-positive cells were observed in the retinal capillaries of the SDT fatty rats in comparison to those in the SD rats. Furthermore, the P/E ratio in the retinal capillaries of the SDT fatty rats (0.42 ± 0.04, *n* = 5) was significantly lower than that of the age-matched controls (0.61 ± 0.01, *n* = 5, *p* < 0.01; Figure 4b). Immunofluorescence analyses were conducted to determine ICAM-1 and VCAM-1 levels in the retinal vessels after trypsin digestion. The retinal vessels of SD rats did not show staining for ICAM-1 or VCAM-1 (Figure 5a). In contrast, the retinal vessels of the SDT fatty rats stained positive for ICAM-1 and VCAM-1 (Figure 5b).

### 3.3. Inflammatory Responses in the Retina of SDT Fatty Rats

Immunostaining for GFAP was performed to determine whether an inflammatory response was induced in the retina of the SDT fatty rats. Faint GFAP staining was observed from the ILM to the ganglion cell layer (GCL) in the retina of SD rats. GFAP staining was observed from the ILM to GCL and from the IPL to INL, indicating the presence of inflammatory response in the retina of the SDT fatty rats (Figure 5c).

DNA microarray analysis was performed to investigate whether global inflammatory cytokines were induced in the retina of the SDT fatty rats.  Table 1 shows that the expression of four genes, *Mcp1*, *Il1b*, *Icam1,* and *Tnf*, was upregulated more than twofold in the retina of the SDT fatty rats relative to that in the retina of the SD rats at 24 weeks of age. Conversely, the expression levels of *Vegfa* and *Il6* were not upregulated in the retina of the SDT fatty rats (Table 1).

**Figure 6:**
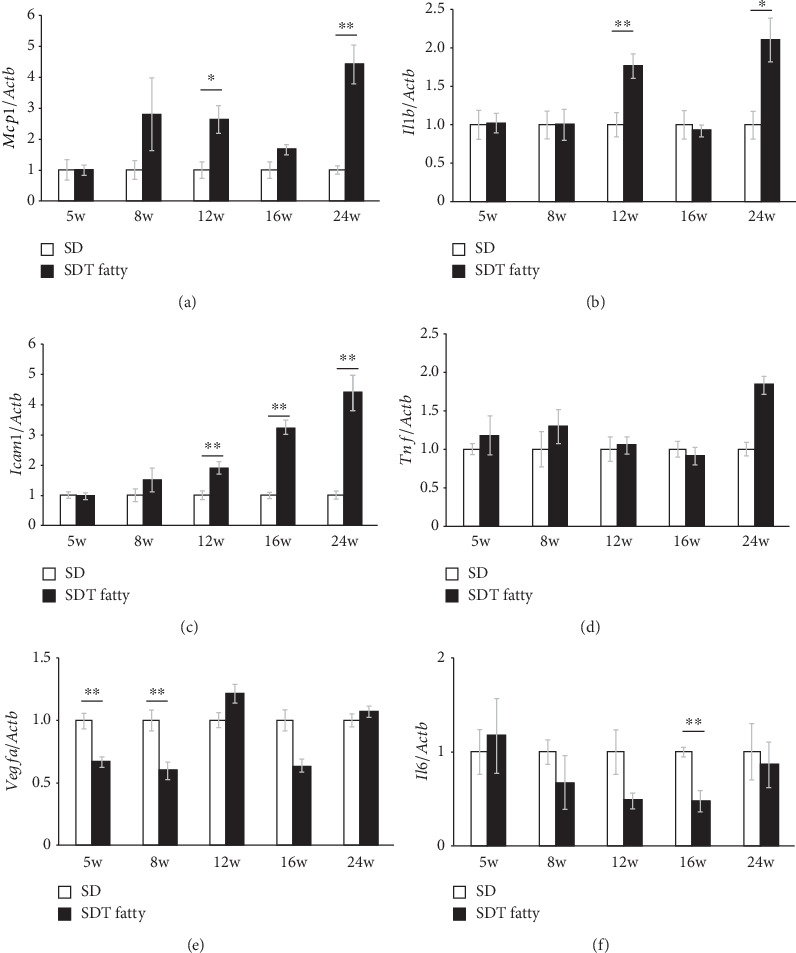
mRNA levels in the retina of SDT fatty rats and SD rats at the age of 5, 8, 12, 16, or 24 weeks. The (a) *Mcp1*, (b) *Il1b*, (c) *Icam1*, (d) *Tnf*, (e) *Vegfa,* and (f) *Il6* levels in the retinal tissues. SDT fatty rats, *n* = 5 each; SD rats, *n* = 4 to 5 each. ⁣^∗^*p* < 0.05, ⁣^∗∗^*p* < 0.01. SDT, Spontaneously Diabetic Torii; SD, Sprague–Dawley.

The mRNA expression levels were quantified using real-time PCR to validate the microarray analysis data. Consistent with the results of microarray analysis, the mRNA expression levels of *Mcp1*, *Il1b,* and *Tnf* were significantly upregulated in the retina of 24-week-old SDT fatty rats (*Mcp1*, *p* < 0.01; *Il1b*, *p* < 0.05; and *Tnf,p* < 0.01) (Figures 6a, 6b, 6d). The mRNA expression levels of *Icam1* gradually increased with age (Figure 6c). The mRNA expression levels of *Vegfa* and *Il6* remained unaltered, which was also in accordance with the microarray analysis results (Figure 6e,f).

**Figure 7:**
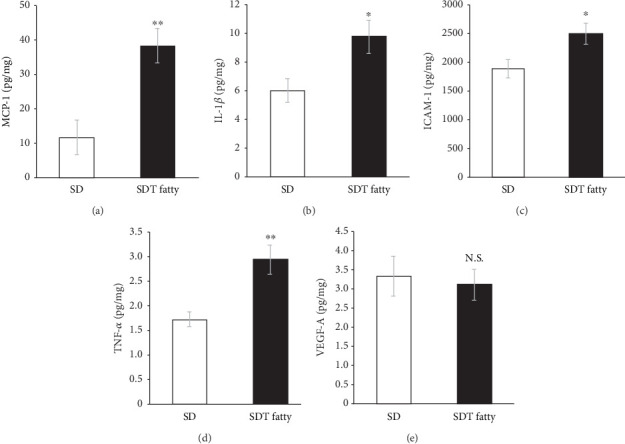
Protein levels in the retina of SDT fatty rats and SD rats at 24 weeks of age. The (a) MCP-1, (b) IL-1*β*, (c) ICAM-1, (d) TNF-*α*, and (e) VEGF-A levels in the retinal tissues. *n* = 5 each. ⁣^∗^*p* < 0.05, ⁣^∗∗^*p* < 0.01. SDT, Spontaneously Diabetic Torii; SD, Sprague–Dawley.

Magnetic Luminex Assay was conducted to evaluate protein expression levels at 24 weeks of age in addition to mRNA. MCP-1 levels showed a significant elevation in the retina of SDT fatty rats in comparison to the retina of the SD rats (SD, 11.60 ± 0.62 pg/mg; SDT fatty, 38.14 ± 7.59 pg/mg, *n* = 5 each *p* < 0.01; Figure 7a). The IL-1*β* levels were significantly elevated (9.70 ± 1.15 pg/mg, *n* = 5, *p* < 0.05) in the retina of the SDT fatty rats compared with those in the retina of the SD rats (6.03 ± 0.83 pg/mg, *n* = 5; Figure 7b). Similarly, ICAM-1 and TNF-*α* levels were also significantly increased in the SDT fatty rats (ICAM-1, 2491.48 ± 179.23 pg/mg, *n* = 5, *p* < 0.05; TNF-*α*, 2.93 ± 0.30 pg/mg, *n* = 5, *p* < 0.01) compared with those in the SD rats (ICAM-11,888.94 ± 160.21 pg/mg, *n* = 5; TNF-*α*, 1.72 ± 0.15 pg/mg, *n* = 5; Figure 7c,d). However, no significant difference was observed in the VEGF-A levels between the SD and SDT fatty rats (Figure 7e). IL-6 levels were below the detection limit in the SD and SDT fatty rats (data not shown).

## 4. Discussion

The present study demonstrated the presence of diabetes-induced retinal changes in the SDT fatty rats. Thickening of all retinal layers, particularly in INL, was found in the SDT fatty rats. In addition, pericyte loss and capillary dilation, which are early changes observed in patients with DR, were identified in the retina of SDT fatty rats. Furthermore, inflammation-associated molecules, such as MCP-1, IL-1*β*, ICAM-1, and TNF-*α*, but not VEGF and IL-6, were elevated in the retina of SDT fatty rats. These findings indicate that the SDT fatty rat is an animal model capable of mimicking early changes observed in patients with DR.

The parafoveal retinal thickness increases with DR progression in humans [7, 8]. The prevalence of DME, which is defined as retinal edema caused by diabetes-induced microvascular complication, increases with the duration of diabetes and progression of DR. [9, 10] Therefore, the cumulative increase in retinal thickening with the progression of retinopathy in humans is presumably due to edematous swelling in the retina. The present study demonstrated that the sensory retina of the SDT fatty rats exhibited increased thickness in comparison to the control animals. Additionally, the retinal edema was not evident in the fixed tissue sections. The increase in tissue thickness was particularly remarkable in the INL of SDT fatty rats. Several studies have reported that an increased retinal thickness is predominantly detected in the INL of patients with DR. [11–13] Postmortem histological analysis of the eyes of patients with diabetes revealed that the majority of microaneurysms originate from the INL [14], indicating the vulnerability of the deep retinal capillary plexus to diabetic vascular complications. Motohashi et al. also reported that the INL of SDT fatty rats is significantly thicker than that of the control SD rats [15]. They also demonstrated that retinal vascular permeability was higher in the SDT fatty rats [15]. Therefore, INL thickening of the SDT fatty rats observed in the previous and current studies may be attributed to retinal edema, which is defined as fluid accumulation that is not apparent in the fixed tissues. The number of pericytes covering the retinal vessels was significantly reduced in the SDT fatty rats in the present study. Pericyte loss is a characteristic sign of early DR and is consistently observed in both humans and animals with diabetes. Pericytes play a crucial role in maintaining the integrity of the inner blood–retinal barriers by surrounding the capillaries and postcapillary venules. The P/E ratios in the retina and lungs are 1:1 and approximately 1:10, respectively [16], indicating the tightly regulated barrier function in the retinal capillaries. Pericyte apoptosis increases endothelial cell permeability in pericyte and endothelial cell cocultures [17]. Thus, retinal thickening in the SDT fatty rats might be attributed to increased vascular permeability caused by pericyte loss. Since pericytes also regulate vascular tone and perfusion pressure [18], pericyte loss might lead to vessel dilation in the retina of the SDT fatty rats. Alternatively, retinal thickening in the SDT fatty rats might be due to mechanisms affecting retinal structure in addition to retinal edema. Tanaka et al. reported retinal folds in the SDT fatty rats. [19] They proposed the hypothesis that the mechanism of retinal fold formation involves changes in retinal volume due to retinal edema and/or proliferation. [19] In the present study, retinal folds and retinal thickening were also observed in the retina of 24-week-old SDT fatty rat. In human, retinal traction by the epiretinal membrane causes retinal thickening and retinal folds. [20] Similar retinal traction may be involved in retinal thickening and folds in the SDT fatty rats, it has been reported that retinal fibrous proliferation and traction were observed in SDT rats at 70 weeks of age, whereas those were not observed in SDT fatty rat up to 40 weeks of age. [5, 21] Further investigation is needed to fully elucidate the underlying mechanism.

To support the mechanism of retinal thickening due to capillary change, the alterations in the capillary diameter of the retina of the SDT fatty rats were evaluated. Retinal capillary dilation is a structural change that is frequently observed in the early stages of DR. Retinal capillaries were dilated in human donor eyes with nonproliferative DR. [22] Furthermore, previous studies using an adaptive optics scanning light ophthalmoscope also revealed that the retinal capillary diameter was increased in patients with nonproliferative and proliferative DR compared with that in healthy controls [23, 24]. In the present study, the retinal capillary diameter in the 24-week-old SDT fatty rats was increased similar to human DR, indicating that the animal model mimics retinal vascular changes that are commonly observed in patients with DR.

The levels of various inflammation-related molecules are elevated in the vitreous of patients with DR. [25–27] Thus, DR has been proposed as an inflammatory disease [28, 29]. ICAM-1 upregulation has been observed in the retinal vessels of patients with diabetes [30]. MCP-1 is a potent chemotactic factor that can alter retinal vascular permeability [31]. IL-1*β* is produced by endothelial cells and microglia under hyperglycemic conditions, and it causes pericyte apoptosis through NF-*κ*B activation and increases endothelial cell permeability [32]. The increase in TNF-*α* expression induces pericyte apoptosis via the downregulation of AKT/p70S6 kinase signaling [33]. Müller cell activation in diabetes is well established. Enhanced expression of GFAP has been reported in the retinal tissue of patients with no or mild DR. [34, 35] Increased expression of inflammation-related molecules and leukocyte adhesion molecules and enhanced expression of GFAP in the retina were observed in the present study, indicating that diabetes-induced inflammatory changes occur in the retina of SDT fatty rats. The inflammatory-related molecules increase the permeability of retinal blood vessels. In addition, Müller cells play an important role in regulating retinal blood flow and maintaining the blood–retinal barrier. [36, 37] It has also been reported that GFAP is increased in the aqueous humor of human DME patients. [38] The inflammatory changes that occur in the retina of SDT fatty rats may contribute to the aforementioned retinal thickness.

In contrast, VEGF-A and IL-6 levels, which were elevated in the vitreous of patients with DR [4, 39], were not elevated in the retina of SDT fatty rats up to 24 weeks of age. Thus, it is possible that retinal changes in the 24-week-old SDT fatty rats correspond to those observed in humans during the early stages of DR, particularly preischemic changes.

The present study has some limitations. First, retinal capillary diameter and vascular permeability were not assessed *in vivo* as SDT fatty rats developed severe cataracts as they grew, and fundus examination was technically impossible. Yu et al. reported a methodology for examining vascular permeability using retinal flat mounts, a technique that might evaluate vascular leakage in SDT fatty rats, which could not be determined in the present study [40]. Second, microaneurysms, another early change observed in patients with DR, were not observed in the trypsin-digested retinal specimens of 24-week-old SDT fatty rats. An et al. demonstrated no association between the increase in capillary diameter and the number of microaneurysms in human donor eyes with non-proliferative DR. [22] The data accumulated in previous studies and the present study indicate that retinal capillary dilation is a prerequisite for microaneurysm formation in patients with DR. However, microaneurysms may develop in older SDT fatty rats as microaneurysms were found at older ages in other diabetic rat models, such as streptozotocin (STZ)-induced diabetic models and Otsuka Long-Evans Tokushima fatty rats [41–43]. Additional investigation is warranted to further elucidate the details of retinal vascular changes in SDT fatty rats.

Rodents have been utilized to study the pathogenesis of DR; however, their use is associated with several complications. The STZ-induced diabetic model has been extensively used for investigating the pathophysiology of diabetes and its complications. However, the onset observed in the model mimics that of Type 1 diabetes; thus, the model may be unsuitable for researching Type 2 diabetes. As spontaneous Type 2 diabetes models, the OLETF and SDT rats show late onset of diabetes at 18 weeks [44] and after 20 weeks [21], respectively; thus, these models require a long study period and are associated with increased costs. In contrast, SDT fatty rats utilized in the present study exhibited hyperglycemia as young as 5 weeks of age [6, 45]. In this study, we showed that SDT fatty rats exhibited inflammation and microvascular changes that are characteristic of the early stages of DR. The use of SDT fatty rats may aid in elucidating the early pathogenesis of DR and developing therapeutic strategies.

## 5. Conclusions

Our findings suggested that SDT fatty rats exhibited early diabetic changes in the retina and indicated that SDT fatty rats could be a potential animal model for the research on the pathogenesis of early human DR.

## Figures and Tables

**Table 1:** Inflammatory cytokine expression in the retina of SDT fatty rats.

**Gene Symbol**	**SD signal (control)**	**SDT fatty signal**	**Ratio**	**GenBank Accession**
*Mcp1*	4.1	66.3	16.13	NM_031530
*Il1b*	16.6	56.5	3.40	NM_031512
*Icam1*	293.0	952.5	3.25	NM_012967
*Tnf*	24.3	60.1	2.47	NM_012675
*Vegfa*	10602.8	11077.9	1.04	NM_001287107
*Il6*	2.9	2.3	0.80	NM_012589

## Data Availability

The data that support the findings of this study are available from the corresponding author upon reasonable request.
